# Spatiotemporal distribution and evolution pattern of Chinese Go League clubs in 20 years of professionalism

**DOI:** 10.3389/fspor.2023.1061751

**Published:** 2023-03-06

**Authors:** Chen Kunlun, Liu Xiaoqiong, Zhang Xu, Ding Lei

**Affiliations:** ^1^ School of Sports, China University of Geosciences (Wuhan), Wuhan, China; ^2^ China Institute of Mountaineering and Outdoor Sports, Wuhan, China; ^3^ School of Environmental Studies, China University of Geosciences (Wuhan), Wuhan, China; ^4^ School of Resource and Environment Engineering, Wuhan University of Technology, Wuhan, China; ^5^ Research Centre for Industrial Economics around Hangzhou Bay Region, Ningbo Polytechnic, Ningbo, China

**Keywords:** Chinese Go League, Chinese Go clubs, host city, spatiotemporal analysis, professionalism

## Abstract

Over the course of 20 years, from 1999 to 2018, the Chinese Go League has gradually developed and flourished, becoming a significant part of urban sports culture. Using mathematical statistics, spatiotemporal trajectory, and geospatial analysis, this research investigates the spatiotemporal distribution pattern and diffusive evolution characteristics of Chinese Go League clubs from the perspective of sports geography. The results of this study show that the following: (1) Over the past 20 years, the number of Chinese Go League clubs has increased in a “stepped” pattern, and there is still plenty of room for expansion. (2) Chinese Go League clubs are primarily located in municipalities directly under the central government and provincial capital cities (accounting for 82.6% of the total), with the Bohai Rim, Yangtze River Delta, and Southwest region of China forming a spatial pattern. (3) Host cities of Chinese Go League clubs are generally discrete, with clustering and random distribution occurring only seldom. The spatial distribution also tends to favor southern cities. (4) Chinese Go League clubs frequently change their names and host cities, and they have a variety of links with those cities, which primarily includes urban stability, urban transition, and urban fluctuation. In future, the professionalization and sustainable development of Chinese Go industry can be enhanced by the marketability of Chinese Go clubs, developing new markets for Chinese Go, and building a healthy development model with multiple linkages of Chinese Go clubs, sponsors, and host cities.

## Introduction

Chinese Go was created in China in 2356 BC and has been around for 4,000 years, according to the Encyclopedia Britannica and the Encyclopedia Americana. The concept of Chinese Go has served as the foundation for Chinese philosophy and culture and is ingrained in Chinese people's cultural heritage. Chinese Go, one of the four old Chinese arts (Lyre-playing, Go, Calligraphy, and Painting), is regarded as a “treasure of Chinese culture” and a crucial means of passing down traditional Chinese culture. It is also a unique sport that blends culture and competitiveness (1). With its rich cultural implications and wide market potential, Chinese Go has become a typical representation of the fusion of culture and sports sector thanks to its professional development (2). Since the founding of the Chinese Go League in 1999, Chinese Go has been professionally organized, which has not only drawn market interest and investment but also accelerated the growth of this sport event with both cultural and sporting characteristics. The imperfect professional sports system, the unequal distribution of clubs, and the relatively small overall influence of the Chinese Go market are just a few of the issues that need to be taken seriously despite the fact that Chinese Go has made significant progress toward industrialization. The issues pose challenges for Chinese Go's continued development (3). Therefore, a scientific analysis of the spatiotemporal distribution and diffusion process of Chinese Go clubs in the spirit of “cultural confidence” and with a reasonable regional distribution guideline are not only one of the key points for the professionalization reform of Chinese Go (4) but also one of the key routes to increase its socioeconomic stickiness and influence.

Sports have deep ties to location, a repeating issue in both sports and human geography that has not gotten enough attention. The spatial distribution and driving forces behind the spread of sports culture are significant issues that both sports humanities and human geography share (5–8). The spatiotemporal analysis of the growth of professional sports is currently significant and has steadily emerged as one of the key points, particularly for popular sports like soccer and basketball (9–11). Soccer is the most successful commercial sport in the world, and the study on the spatiotemporal characteristics of its club operations, talent selection, and league connections offers a fresh perspective that will help us gain a thorough understanding of the sport's growth patterns (12). Moreover, soccer is also one of the earliest important fields where interdisciplinary research on sports and geography has been done. It has contributed significantly to compiling and demonstrating the development of sports geography in China. Focusing on the territorialization of professional soccer clubs, researchers like Tian Zhimei, Wang Dezhi, and Chen Kunlun have examined the spatiotemporal distribution characteristics, patterns, and influencing mechanisms of soccer clubs (13–15). They have also discussed the phenomena of home field migration and spatial diffusion (4, 16, 17). These studies have deepened popular perceptions of the spatiality of sports culture in addition to expanding the research connotation of sports geography (13, 18). Regarding basketball, academics have focused on the status of development and the phenomena evolution of basketball clubs (9, 19). Other scholars have also examined the spatiotemporal distribution traits and driving factors of Olympic champions and national bases of the sports sector (20, 21). These studies have advanced the interdisciplinary integration of sports and geography and the development of sports geography by utilizing the traditional spatiotemporal analytic approach of geography.

The economic and cultural aspects of Chinese Go are currently the main topics of relevant studies. The industrialization of Chinese Go (2, 22) and the professionalization of the Chinese Go League (3, 23) are subjects of great interest from an economic perspective. These studies offer suggestions and references to illuminate and direct the increasing commercialization and industrialization of the Chinese Go League. Chinese Go is recognized as a representative of traditional Chinese sports culture from a cultural standpoint, and its historical significance and current mission have importance, such as cross-cultural communication benefits of the Chinese Go League (24–26). The viewpoints of sports management, sports culture, and sports communication are primarily used in related studies. A new window for understanding sports phenomena is provided by sports geography, and the spatiotemporal distribution and diffusion patterns of Chinese Go clubs will offer a fresh viewpoint on the in-depth knowledge of the professional development of Chinese Go. In light of this, this research investigates the spatiotemporal distribution and diffusive evolution of Chinese Go League clubs from the perspective of sports geography, utilizing the methods of mathematical statistics, spatial agglomeration model, and spatiotemporal trajectory. In an effort to clarify the relationship between clubs and cities and provide references for the professionalization of Chinese Go and the growth of related sports and cultural sectors in China, this study aims to summarize the spatiotemporal evolution pattern of clubs in the 20 years of professional development of Chinese Go from 1999 to 2018.

## Research design

### Research subject

Since its inception in 1999, the Chinese Go League, the top-tier league in Chinese Go, has served as a significant arena for Chinese Go clubs. Since the 2001 season, Chinese Go leagues have started implementing promotion and relegation procedures. The bottom two teams in League One are demoted to League Two at the end of each season, and the top two teams in League Two are promoted to League One. Since the 2017 season, the Chinese Go League has expanded to 14 teams, with three more teams eligible for promotion and relegation. This research focuses on the 20 years of Chinese Go's professionalization from 1999 to 2018 and takes into account all of the clubs (teams) competing in the Chinese Go leagues (Table 1).

**Table 1:** Related data of Chinese Go First League and Chinese Go clubs, 1999–2018.

Year	Club number	Province number	Number of main cities	Chess player number	League sponsor
1999	10	9	10	59	Jiangling
2000	12	10	11	71	Jiangling
2001	12	10	10	73	Jiangling
2002	12	11	11	73	Jiangling
2003	12	10	10	73	Good Cats
2004	12	9	10	70	—
2005	12	9	10	71	Outride
2006	12	8	9	71	China
2007	12	10	10	71	Gionee
2008	12	10	10	68	Gionee
2009	12	11	11	70	Gionee
2010	12	11	11	68	Gionee
2011	12	10	11	68	Gionee
2012	12	9	11	67	Gionee
2013	12	10	12	70	Gionee
2014	12	11	12	71	Gionee
2015	12	10	10	70	Gionee
2016	12	10	10	68	Gionee
2017	14	11	12	84	Gionee
2018	14	10	11	102	Huawei

### Data source

The statistical data related to Chinese Go League clubs are sourced from the official website of the Chinese Go League, official website of the Chinese Weiqi Association, official websites of local chess institutes, related news reports, professional sports websites (NetEase, Sohu, Sina Sports), and official websites of clubs. The “team/time” is used as the unit of record in order to scientifically reflect the relationship between Chinese Go clubs and host cities, meaning that if one club from a city enters the Chinese Go League during a certain season, it is recorded as 1 team/time, 2 teams/time for the entry of two teams, and so on.

The WorldMap platform is primarily used to collect geographic data, which is then entered for further processing into the ArcGIS10.0 program. The administrative map of China is used as the starting point in this study's attempt to scientifically mark the spatial attributes of Chinese Go League One clubs. The WorldMap platform is used to obtain the club's precise coordinates, which are then set as point targets in ArcGIS10.0, matched to specific cities, and given relevant attributes. This creates the spatial distribution database of Chinese Go League clubs.

### Research methodology

This paper integrates the methods of mathematical statistics and geospatial analysis. Mathematical statistics is used to quantitatively describe the spatiotemporal distribution characteristics of Chinese Go League clubs, and the geospatial analysis methods such as spatial agglomeration model and spatiotemporal trajectory are utilized to reveal the spatiotemporal distribution patterns and diffusive evolution processes of Chinese Go League clubs.

#### Spatial agglomeration model

The nearest neighbor point analysis of the ArcGIS10.0 platform is mainly applied to calculate the nearest neighbor index of Chinese Go League clubs (8), and then analyze the aggregation of its spatial distribution with the following calculation formula:

$$d(NN)=\sum_{i=1}^{N}\left[\frac{Min(d_{i})}{N}\right]\;d(\mathrm{ran})=0.5\sqrt{\frac{A}{N}}NNI=\frac{d(NN)}{d(\mathrm{ran})}$$

where *d*(*NN*) represents the mean value of the shortest distance from each point to its nearest point, Min(*d_i_*) is the distance from a point to its nearest point, and *N* is the number of samples; *d*(ran) denotes the expected value of the nearest distance in the case of completely random distribution, *A* is the area of the study area, and the nearest neighbor index *NNI* means the ratio of the observed nearest distance to the average distance in the random case. Theoretically, the value of *NNI* ranges within 0–2.1491. When *NNI* is close to 1, it indicates that the spatial distribution of geographic phenomenon is random; when *NNI* < 1, it indicates that the geographic phenomenon shows a state of aggregation, and the smaller the value, the stronger the aggregation; when *NNI* > 1, it is dispersed, and the larger the value, the stronger the dispersiveness.

#### Spatiotemporal trajectory

**Figure 1:**
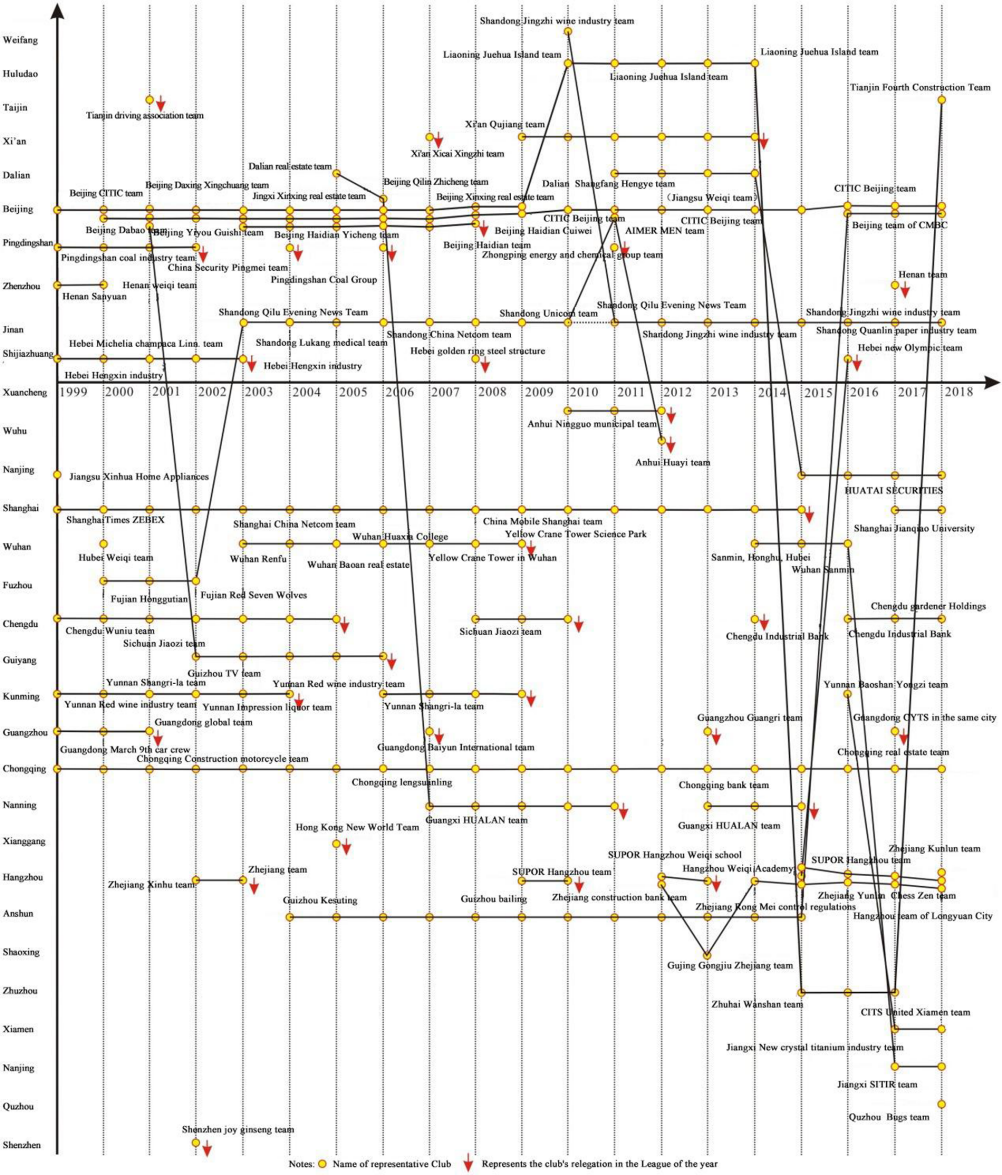
Spatiotemporal trajectory of Chinese Go clubs.

The spatiotemporal trajectory, one of the important means to realize the visualization of spatiotemporal characteristics (14), represents the time and geographic space by a horizontal two-dimensional coordinate and connects an object with lines like the spatiotemporal movement trajectory of a person (27, 28), where the vertical coordinate denotes the time and the horizontal coordinate indicates the geographic space. This research constructs a spatiotemporal trajectory of Chinese Go League clubs (Figure 1) with the time axis as the *x*-axis and the distribution cities as the *y*-axis (the upper part of the horizontal axis shows cities in northern China and the lower part shows cities in southern China), for analyzing the spatiotemporal distribution and distributive migration.

## Time series changes of Chinese Go League clubs

### Development process of Chinese Go League clubs

A universal consensus has emerged about the professionalization and marketization of competitive sports (3). China now has relatively developed professional league systems in place for the sports of Chinese Go, basketball, ping pong, and soccer. Chinese Go has garnered little attention from the Olympic Games, European, or American nations while being a distinctive “nonmainstream” sport in East Asian culture. However, it has offered East Asia good commercial prospects and development potential since its inception. The “20 years of Chinese Go League” could be divided into the Jiangling period (1999–2002), volatility period (2003-2006), including Good cats and Outride, the Gionee period (2007–2017), and the Huawei period (2018) based on the names of the league (Table 1).

Chinese Go League clubs are becoming more numerous in a leisurely, “stepped” fashion (Table 1). Chinese Go League clubs have seen a transition over the past two decades, moving from a challenging situation of not having enough clubs to one of the heated competitions for promotion and relegation. The league was supposed to have 12 teams in its inaugural season in 1999, but only 10 clubs actually participated because the Shanghai 2nd Team and Zhejiang Team were unable to get sponsorships. This terrible situation served as a stark reminder of how tough it was to professionalize Chinese Go in its early stages, when there was little market acceptance. Even after five years of operation, in 2004, this circumstance recurred. The Chinese Go League has since grown and expanded progressively, with 12 clubs continuing to compete from 2000 to 2016. With the addition of 14 teams in the 2017 season and an increase in the number of players on the front line that reached over 100 in the 2018 season, the Chinese Go League has entered a state of expansion. In the 2019 season, the Chinese Go League saw yet another increase in clubs, bringing the total to 16.

Chinese Go clubs also grew as the league became more professional, much like soccer. Chinese Go was a government-funded social project during the time of the planned economy. Chinese Go began the market-oriented reform and the introduction of the club system in 1999, which paved the way for professionalization, in order to meet the needs of the market economy and the needs of the people for sports culture after the reform and opening up. Under the direction of the Chinese Weiqi Association at the time, Chinese Go clubs were transformed from sports teams (local chess institutions), and their market awareness, economic vitality, and competitive capacity were not adequately pushed and unleashed. Chinese Go clubs (teams) were urged to give up their naming rights for financial gain in order to support the market orientation. The two early teams in Chongqing and Shanghai were typical instances of Chinese Go clubs whose names frequently changed due to changes in sponsorship. The nine-time champion team from Chongqing has gone by a variety of names over the years, including Chongqing Jianshe Motorcycle, Chongqing Lesening, Chongqing Bank, Chongqing Real Estate, and Chongqing Aip Real Estate. The Shanghai team has also gone by a variety of names over the years, including Shanghai Shidai Jusheng, Shanghai Mobile, China Mobile Shanghai, and Shanghai Jian Qiao University. In the early years of the league, corporate sponsorship was the Chinese Go League clubs’ primary source of income. For instance, Chongqing Construction Group, a sizable state-owned corporation, funded the Chongqing Jianshe Motorcycle Club. In 1991, Beijing Sanlu Factory (Beijing Dabao Cosmetics Co., Ltd.) and Beijing Chess Institute collaborated to create the Beijing Dabao Team. In addition, more than one business participated in the sponsorship. For example, Chengdu Weiqi Federation and eight companies collaborated to form Chengdu Wuniu Club (Sichuan Jiaozi Team).

Of course, some clubs had consistent sponsorship from the same companies, keeping their names consistent. For example, Guizhou Bailing Pharmaceutical Co., Ltd, founded the first and only Chinese Go team, the Guizhou Bailing Team. Nevertheless, because of the strong cultural legacy, few teams’ names changed despite the changes of multiple investors. Beijing Haidian Team, for instance, has gone by the names Beijing Haidian Yicheng, Beijing Haidian Sohu-AMD, and Beijing Haidian Cuiwei, but the name “Beijing Haidian” was kept, and the team's members and culture from the previous Beijing Haidian Chess Institute were kept.

In conclusion, the professionalization and marketization of Chinese Go clubs have advanced along with the socialist market economy system's ongoing reforms, with the number of clubs gradually rising over the past two decades (Figure 1), experiencing semiprofessional to professional development, and gradually diversified club sponsors or investment bodies, while club development has shifted to emphasize cultural connotations and brand continuity. The market flexibility and inclusivity of the development of Chinese Go have been expanded, and the degree of commercialization and professionalization has increased, on the one hand, pushed by the logic of market economy development (29). A vast industrial chain related to Chinese Go has emerged as a result of the commercialization and market-driven professionalization of the Chinese Go tournament, which has had a positive impact on the growth of the sports and cultural industries and produced significant economic and social benefits (30). The “Healthy China” plan and the sustained growth of China's social, economic, and cultural development are both supported by this.

### Urban distribution of Chinese Go League clubs

The distribution of Chinese Go League clubs in each city has changed dramatically and unevenly during the past 20 years. There were multiple clubs playing in the same season in some cities. In the early period of the league (2006), Beijing had four Chinese Go League clubs: Beijing Xinxing Real Estate, Beijing Dabao, Beijing Haidian Sohu-AMD, and Beijing Kirin Zhicheng; in the midterm of the league (2015), Hangzhou had three Chinese Go League clubs: Supor Hangzhou, Zhejiang Yunlin Chess Zen, and Hangzhou Chess Institute; and recently (2018), Hangzhou had three clubs, namely, Supor Hangzhou, Zhejiang Kunlun, and Longyuan Future City Hangzhou. Simultaneously, some host cities, such as Tianjin, Shenzhen, Shaoxing, Wuhu, and Weifang, which each had just one club that briefly entered the Chinese Go League in one or two seasons, were also short-lived. Chinese Go League clubs are primarily concentrated in municipalities like Beijing, Chongqing, Shanghai, and provincial capital cities, such as Hangzhou, Jinan, Chengdu, Wuhan, etc., according to statistics on the frequency of host cities (31). A strong basis for the development of Chinese Go League clubs can be created by the healthful market environment in large cities (Table 2).

**Table 2:** Frequency and first year of home city of Chinese Go clubs, 1999–2018.

City	First appearance (year)	Always frequency	City	First appearance (year)	Always frequency	City	First appearance (year)	Always frequency
Beijing	1999	42	Shijiazhuang	1999	7	Tianjin	2001	2
Chongqing	1999	20	Xi’an	2007	7	Xiamen	2017	2
Shanghai	1999	19	Guangzhou	1999	6	Nanchang	2017	2
Hangzhou	2002	18	Nanjing	1999	5	Shenzhen	2002	1
Jinan	2003	16	Guiyang	2002	5	Hong Kong	2005	1
Chengdu	1999	14	Dalian	2005	5	Weifang	2010	1
Anshun	2004	12	Huludao	2005	5	Wuhu	2012	1
Kunming	1999	11	Zhengzhou	1999	3	Shaoxing	2013	1
Wuhan	2000	11	Fuzhou	2000	3	Quzhou	2018	1
Nanning	2007	8	Xuancheng	2010	3			
Pingdingshan	1999	7	Zhuhai	2015	3			

## Spatial distribution characteristics of Chinese Go League clubs

### Overall pattern of Chinese Go League clubs

Chinese Go League clubs are typically spilt between 9 and 11 host provinces during a single season (Table 1); however, 2006 was an outlier as there were only 8 provinces. The Chinese Go League started to grow in 2007 when two new provinces, Guangxi (Hualan Team) and Shaanxi (Xicai Zhixing Team), were included. With Jiangxi (Xinjing Titanium Team) added in 2017–2018, this pattern persisted. There are currently no Chinese Go League clubs in 10 provinces, including Hunan, Shanxi, Inner Mongolia, Heilongjiang, Jilin, and Hainan.

Chinese Go League clubs are primarily distributed in provincial capital cities (48.3% of the total) and municipalities directly under the central government (34.3% of the total) in terms of host city classifications (Table 2). In comparison to soccer clubs in similar cities (77.6%), their overall share (82.6%) is higher. This indicates that the economic, political, and cultural variables have a significant impact on Chinese Go as well. The development of Chinese Go League clubs can be better supported by municipalities directly under the central government, provincial capital cities, or other mega-cities with larger and higher rankings since they have a wider market and longer cultural accumulating of Chinese Go. For instance, Hangzhou, which has recently had great success, has maintained two or three Chinese Go League clubs since the 2012 season. It is the outcome of collaboration and ongoing investment by professional institutions and business entities like the Hangzhou branch of the China Chess Institute and Supor. In addition, there are a few second and third tier cities with well-known Chinese Go League clubs, such as clubs established in Pingdingshan in Henan Province and Anshun in Guizhou Province, which relied on the support of the Pingmei Group and the Bailing Group, respectively. Although Pingmei Club is struggling on the brink of promotion and relegation, it still has importance to the club and invests in it continuously. Prior to being acquired by the China Minsheng Bank and relocating to Beijing in 2016, the Guizhou Bailing Club had been performing well (winning runners-up in seven seasons) since joining the Chinese Go League in 2004. The importance and love that Pingdingshan and Anshun have for the Chinese Go sport and its culture have been evident.

Chinese Go League clubs exhibit a clearly unbalanced spatial distribution in accordance with the regional development pattern (Table 3), with the greatest concentration in the east, followed by the west, and the least in the central region. Chinese Go League clubs are primarily located on China's eastern coast, making up around 57.0% of the country's total clubs. Chinese Go League clubs are even distributed in cluster across specific provinces and cities (Beijing and Zhejiang). For example, Zhejiang had four clubs for the 2018 season, three of which were in Hangzhou: Supor Hangzhou, Longyuan Future City Hangzhou, Zhejiang Kunlun, and Quzhou Bagesi Team. The clubs in Chongqing, Chengdu, and Kunming have maintained a good continuity by participating in the Chinese Go League for years with a frequency accounting for 31.8% of the national total, despite the fact that certain provinces in the western area have never had Chinese Go League clubs. It is impossible to overlook how strong sports culture is in the western region in China. The central region has the fewest clubs. Other than Wuhan, most cities’ Chinese Go League clubs are entrapped in a condition of instability. It is noteworthy that the degree of distributing imbalance in Chinese Go is relatively good, in contrast to the spatial characteristics of host cities for soccer.

**Table 3:** Each region's quantity and ratio change of Chinese Go clubs, 1999–2018 (unit: home/time).

	1999–2002		2003–2006		2007–2017		2018		Whole	
	Quantity	Proportion (%)	Quantity	Proportion (%)	Quantity	Proportion (%)	Quantity	Proportion (%)	Quantity	Proportion (%)
Eastern cities	26	56.5	25	52.1	76	52.8	11	78.6	138	57.0
Central cities	7	15.2	6	12.5	13	9.0	1	7.1	27	11.2
Western Cities	13	28.3	17	35.4	45	31.2	2	14.3	77	31.8

**Figure 2:**
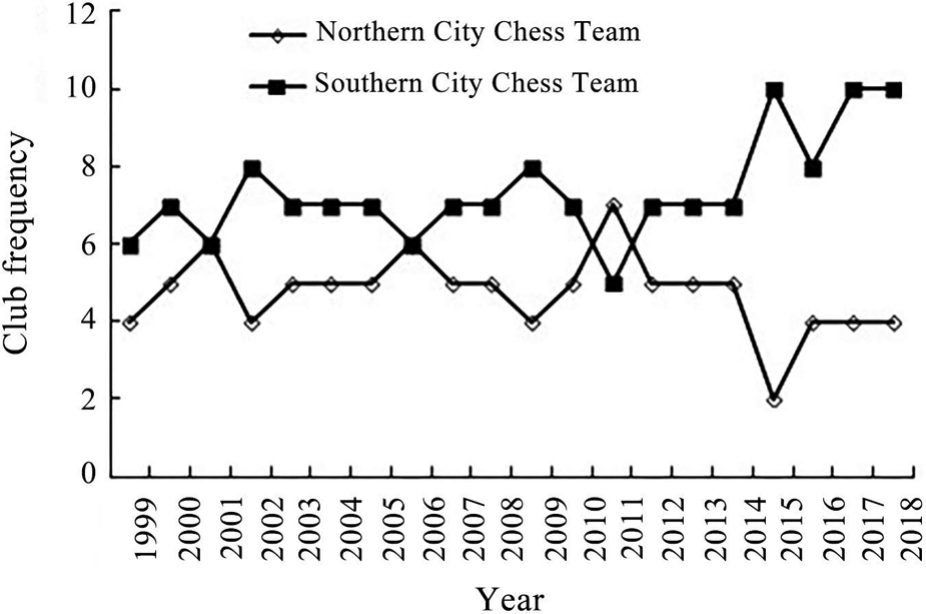
Comparison of the distribution of Chinese Go clubs between the north and the south.

In the light of the north–south geographical pattern (Figure 2), the spatial distribution of Chinese Go League clubs favors the south, with only 10 clubs in cities to the north of the “Qinling-Huaihe” line, but as many as 21 clubs in cities to the south. With the exception of 2011, there were fewer Chinese Go League clubs in northern cities than in southern ones in every season. The gap between northern and southern cities grew even wider, especially after 2015, with just 4 in northern cities and 8, 10, and 10 in southern cities from 2016 to 2018. Relegation occurred 12 times (12.6%) in northern cities and 19 times (12.9%) in southern cities, which indicates that there are no appreciable differences in the continuity and stability of Chinese Go League clubs between northern and southern cities. This characteristic is distinct from that of soccer clubs because, in terms of quantity, stability, and continuity, soccer clubs in northern cities are undoubtedly superior to those in southern ones. The Chinese Go culture is more widespread in southern cities where the Chinese Go market is more developed and there is a large pool of professional players, despite Beijing, where the China Chess Institute is located, being the most significant cultural center of Chinese Go and holding an absolute dominant position in the total frequency of clubs (42 clubs/time, 17.4%). The sponsors of the Chinese Go League and vital competitions (such as the Chunlan Cup, Bailing Cup, and Ing Cup) are also primarily southern Chinese enterprises.

### Spatial agglomeration of Chinese Go League clubs

As far as the spatial relationship is concerned (Table 4), the spatial distribution of Chinese Go League clubs is generally discrete, indicating that the spatial interaction of host cities is poor. Agglomeration and random dispersion occur occasionally, although there is generally little discernible radiation influence across cities. There are numerous clubs in Shanghai and Hangzhou, but Jiangsu and Jiangxi in the vicinity only have Chinese Go League clubs in capital cities (five times in Nanjing and two times in Nanchang). Hunan and Shanxi, for instance, do not have Chinese Go League clubs, but their neighboring provinces have maintained Chinese Go League clubs for a long time. A Chinese Go League club from Jiangsu had to move to Dalian, Liaoning Province, because it was unable to secure sponsorship, despite Jiangsu being an economically developed province (2011 season).

**Table 4:** The average nearest neighbor index and related data of Chinese Go clubs, 1999–2018.

Year	Average observation distance (km)	Expected average distance (km)	*NNI*	*Z*-score	*P-*value	Agglomeration characteristics
1999	334.3	220.9	1.512	3.101	0.002	Dispersed
2000	235.6	192.5	1.224	1.484	0.137	Dispersed
2001	309.9	223.5	1.386	2.561	0.010	Dispersed
2002	280.4	223.5	1.254	1.684	0.092	Dispersed
2003	229.2	204.1	1.123	0.814	0.416	Random
2004	231.5	201.8	1.147	0.975	0.329	Random
2005	313.9	223.8	1.402	2.668	0.007	Dispersed
**2006**	**182**.**8**	**201**.**8**	**0**.**863**	**−1**.**005**	**0**.**413**	**Gather**
2007	346.4	203.3	1.703	4.664	0.000	Dispersed
2008	298.7	203.2	1.469	3.114	0.002	Dispersed
2009	284.7	203.9	1.396	2.625	0.008	Dispersed
2010	284.3	193.9	1.466	3.088	0.002	Dispersed
2011	268.7	206.9	1.299	1.981	0.048	Dispersed
2012	225.9	199.4	1.132	0.879	0.379	Random
2013	283.9	221.6	1.281	1.864	0.062	Dispersed
2014	314.8	193.8	1.624	4.136	0.000	Dispersed
2015	261.6	189.5	1.381	2.522	0.012	Dispersed
2016	269.2	205.9	1.307	2.036	0.042	Dispersed
2017	203.8	191.6	1.064	0.457	0.647	Random
**2018**	**165**.**6**	**192**.**8**	**0**.**859**	**−1**.**009**	**0**.**318**	**Gather**

NNI, nearest neighbor index. The two bolded columns show the values for the two years in which there is a clustered distribution of Chinese Go League clubs.

**Figure 3:**
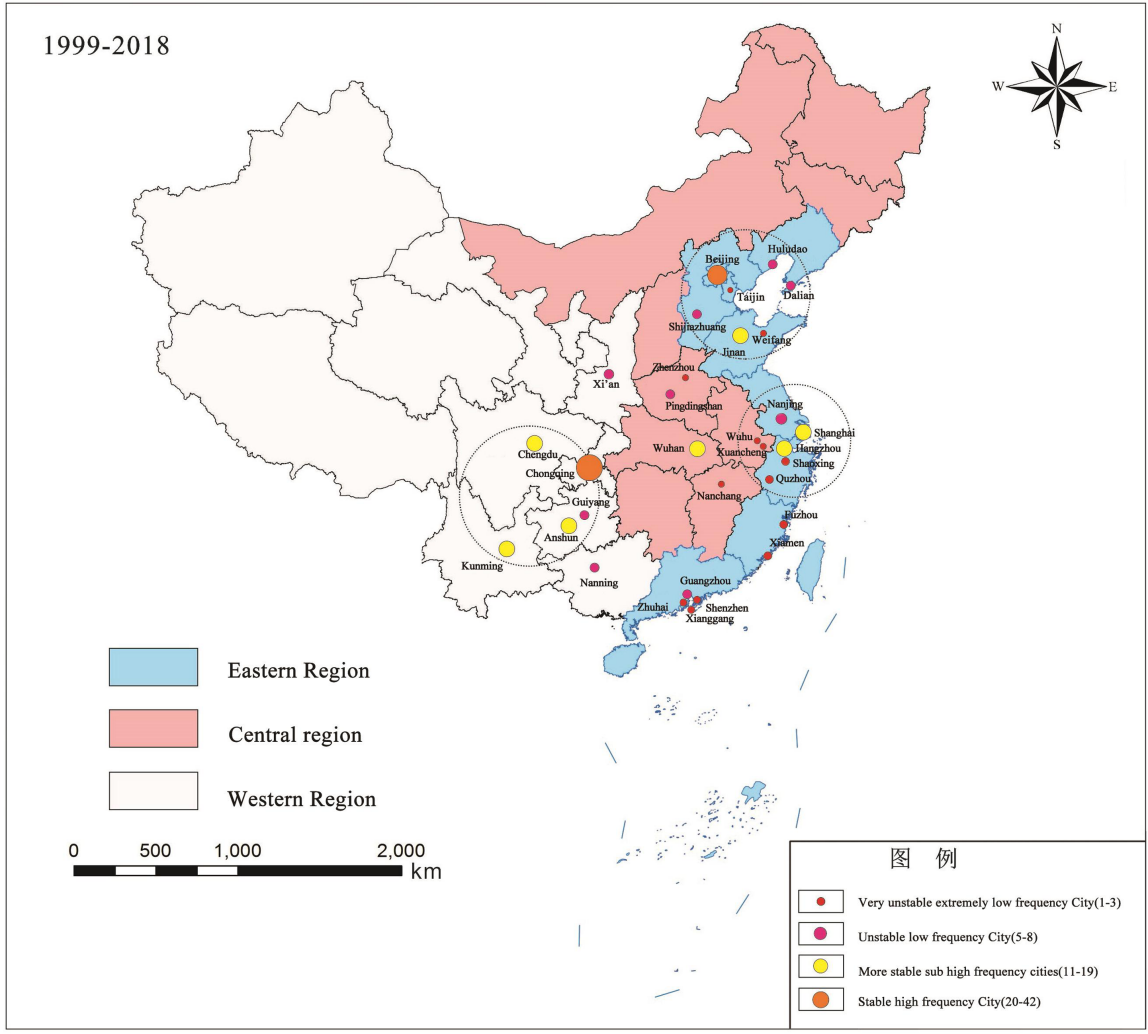
Frequency of home city distribution of Chinese Go clubs, 1999–2018.

By spatial pattern, Chinese Go League clubs have steadily shaped three key clustering zones (Figure 3): the Bohai Rim with Beijing at the core; the Yangtze River Delta with Shanghai and Hangzhou at the core; and the Southwestern Region with Chongqing and Chengdu at the core. Compared to the club hubs that surround the six traditional soccer cities (Beijing, Shanghai, Guangzhou, Wuhan, Dalian, and Chengdu), this is very different. There is no clear center of Chinese Go League clubs, and Wuhan is the only city that consistently hosts Chinese Go League clubs on a regular basis. Additionally, there is no clustering in the Pearl River Delta region, where clubs frequently changed their names in addition to being relegated multiple times (such as Guangzhou Team).

### Spatial diffusion of Chinese Go League clubs

Chinese Go League clubs’ migration and spatial distribution are discontinuous (Tables 1 and 3), lacking the hierarchical diffusion connected to the urban hierarchical order. Tian Zhimei pointed out that the dissemination process of Chinese soccer clubs’ professionalization is hierarchical and based on the concept of proximity, with key cities serving as the source and the size of the cities serving as the order. Chinese Go League clubs’ geographic dispersion is both hierarchical from major cities (Guizhou Satellite TV Club migrated from Beijing Nie Weiping Go Academy, for instance) and convergent to major cities (for example, Guizhou Bailing moved to Beijing and became Minsheng Bank Club). Under the influence of sponsorship and celebrity effects, they show the characteristics of long- and medium-distance cross-regional migration. Typical examples include the Nie Weiping Go Academy and Gujinggong Liquor Zhejiang Team. The primary players of the former team, Nie Weiping and his followers, frequently relocated among five cities, while the latter's migration of host cities (from Hangzhou to Shaoxing in 2013) was connected to the head coach Ma Xiaochun's social network connections with his hometown Shaoxing.

**Figure 4:**
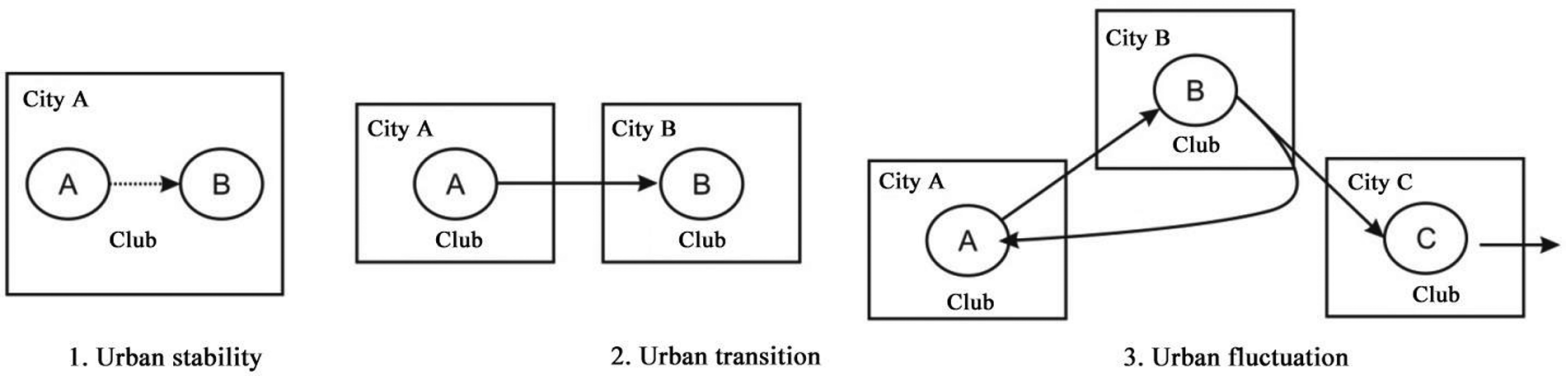
The distribution of Chinese Go clubs and the relationship between clubs and home cities.

Chinese Go League clubs can be divided into three types according to whether clubs migrated and how frequently they traveled (Figure 3), which illustrates the variety of relationships between clubs and host cities (Figure 4):

(i) Urban stability type: Chinese Go League clubs that have never migrated their host cities fall under this category. The Chongqing Team is a typical representative since Chongqing has served as its host city consistently over the course of the “20 years of Chinese Go League,” providing stability and continuity. The Shanghai team mostly maintains the stability but was demoted in the 2015–2016 seasons. These clubs’ names have changed naturally as a result of the change of sponsors, but their host cities remain the same.
(ii) Urban transition type: This category consists of Chinese Go League clubs that have only moved their host cities once. 2015 saw the relocation of Guizhou Bailing Club from Anshun to Beijing and the change of its name to Minsheng Bank Beijing Team. Since being renamed Shandong Qilu Evening News Team after being purchased by Qilu Evening News Go Academy in 2003 and changing its name from Fujian Red Sept Wolves Team, Fujian Team has remained in Jinan. After Honghu Sanmin Culture Communication Co., Ltd, purchased Tianjin University Team and collaborated with Nie Weiping Go Academy, Hubei Honghu Sanmin Team was reorganized and promoted to the Chinese Go League in 2014. It was later relocated to Nanchang and changed its name to Jiangxi Xinjing Titanium Team and Jiangxi Sitir Liquor Team.
(iii) Urban fluctuation type: This category consists of Chinese Go League clubs that have migrated their host cities more than twice. The Beijing Nie Weiping Go Academy migrated most frequently and experienced five different venues in 20 years. Following its successful promotion to the Chinese Go League in the 2001 season, it was named as Beijing Yiyou Guishi Team. For the 2002 season, its home field was moved to Guiyang, Guizhou, where Guizhou Satellite TV Nie Weiping Go Academy was established (relegated in 2006). The “Anhui Ningguo Municipal Go Team” (relegated in 2012) was established in Xuancheng, Anhui Province, in 2010 and was once more elevated to the Chinese Go League. It was renamed Yunnan Baoshan Yongzi Team, and later, in 2016, it was promoted to the Chinese Go League for the third time. However, in the 2017–2018 seasons, its home field was migrated to Xiamen, Fujian Province, and it was renamed as China United Travel Xiamen Team. CITIC Dasanyuan Team, now known as Beijing Xinxing Real Estate Club, also relocated four times. After the 2010 season, it moved to Huludao, Liaoning, and changed its name to Juehua Island Team. The next year, it moved to Zhuhai, Guangdong, and changed its name to Zhuhai Wanshan Team. Finally, it moved to Tianjin in 2018 and changed its name to Tianjin Sijian Team. In 2005, the Hong Kong New World Team that was promoted to the Chinese Go League and Guangxi Hualan Team in Nanjing that was relegated experienced the change of three host cities. A few Chinese Go League clubs have also relocated and returned. Jiangsu Team, for instance, was successfully promoted to the Chinese Go League in 2011 but was unable to secure sponsorship. After being sponsored by Dalian Shangfang Real Estate Co., Ltd., it migrated to Dalian, Liaoning, and changed its name to “Dalian Shangfang Hengye Team.” Its sponsorship changed once more in 2015, as the team relocated back to Nanjing, Jiangsu Province, adopting the moniker “Huatai Securities Jiangsu Team.” Besides Jiangsu Team, the second team of Hangzhou Go Academy is also a typical club that moved away and then back.

Furthermore, there were additional clubs that were sponsored by several sponsors. In 2010, the first team of Shandong Qilu Evening News Club was sponsored by Shandong Qilu Evening News (with the home field in Jinan, winning the championship in that season), and the second team was sponsored by Shandong Jingzhi Liquor (with the home field in Weifang). In the 2011 season, Jingzhi Liquor cooperated with Shandong Qilu Evening News to increase the investment and renamed the first team as Shandong Jingzhi Liquor Team (with the home field in Jinan), and the second team was sponsored by other enterprises and renamed as AIMER MEN Team (with its home field migrated to Beijing). In the 2012 season, the second team was relocated to Wuhu, Anhui Province, for the 2012 season and given the name Anhui Huayi Team; however, sadly, the team was demoted following a disappointing campaign.

Therefore, changes in investors or sponsors are responsible for the migration and spatial distribution of host cities of Chinese Go League clubs. The consistent and steady capital investment is a crucial cornerstone for the reliable running of home grounds, regardless of whether they belong to Chinese Go clubs or capital-light soccer clubs (3). Chinese Go clubs, in contrast, have negligible operational costs and impediments to investors or sponsors. Because it is relatively simple to replace or leave, host cities routinely change sponsors. While studies have indicated that frequent changes in host cities are bad for the Chinese Go culture there, mobility of host cities is favorable for the spread of Chinese Go culture. This will also have an impact on the club's marketing and commercial promotion. Therefore, the policymakers of the sports culture industry and investors of the Chinese Go clubs need to pay attention to how to maintain the stable symbiosis and healthy development of clubs and host cities.

## Discussion

The analysis above led to the following recommendations and countermeasures for the development of Chinese Go League clubs:

- The level of professionalization and standardization of Chinese Go clubs needs to be further improved. In order to speed up their market-oriented operations, reduce the issues of frequent club name changes and host city migration due to unclear property rights, confused management, and inappropriate investment, and gradually establish a long-term and stable brand image of Chinese Go League clubs, it is first necessary to guarantee the independent legal status of Chinese Go clubs.
- The host cities of Chinese Go League clubs changed frequently in most of the seasons. Chinese Go clubs have relatively modest capital investment requirements (far lower than those of soccer and basketball clubs), making it simple to start new clubs or move among cities when promotion and relegation are in effect (31, 32). The diffusion of clubs will increase in new cities as the Chinese Go League expands (there were 16 clubs in the 2019 season). The promotion of Chinese Go and the development of a new market for it will be an extremely practical task at the next level of the league.
- To facilitate the interaction of Chinese Go clubs, capital (sponsors), and host cities, a favorable development model should be created. To maximize reciprocal benefits, the relationships among host cities, capital, and clubs should be examined in order to construct a new, reliable, and effective collaboration and symbiosis model. Chinese Go also possesses the combined attributes of sports and culture, allowing for the establishment of a complete industrial chain that would start with the competition market and be supported by the markets for education, culture, and leisure. This is also where professional and popular Chinese Go will emerge in future.
- Furthermore, as artificial intelligence (AI) and Information Technology (IT) have advanced, the worldwide attention attracted by human and computer play (Korean player Lee Sedol vs. Google AlphaGo in 2016 and Chinese player Ke Jie vs. Google AlphaGo in 2017) has further sparked a trend toward learning Go and promoting the game's culture, opening up new opportunities for Chinese Go League and Go programs. The landscape of the professional league has changed as a result of AI technology, and professional Go club's choice of host cities will be impacted. AI technology has also altered the skill level of professional players (convergence of skills and reduction of the gap in the level of masters). Further studies should pay close attention to each of these difficulties.

## Conclusions

This study investigates the spatial distribution and diffusive evolution of Chinese Go League clubs over the course of the “20 years of Chinese Go League” from the perspective of sports geography, using methods such as mathematical statistics, spatial agglomeration model, and spatiotemporal trajectory. The primary conclusions of this study are as follows:

- The overall number of Chinese Go League clubs has gradually increased in a “stepped” fashion during the past 20 years. Chinese Go and the league market have excellent market prospects with room to grow in future thanks to the professional league's continued development and system improvements. The commercialization and professionalization of Chinese Go League clubs have intensified due to the logic of market economy development, which has also facilitated the growth of the sports and cultural industry as well as the industrial chain related to Chinese Go, resulting in significant economic and social benefits.
- The distribution of Chinese Go League clubs varies noticeably: 82.6% of the total are distributed in provincial capital cities and municipalities directly under the central government. It gradually creates a spatial pattern of the Bohai Rim with Beijing at the core, the Yangtze River Delta with Shanghai and Hangzhou at the core, and the Southwestern Region with Chongqing and Chengdu at the core. The spatial distribution of Chinese Go League clubs favors southern cities.
- Chinese Go League clubs are unequally distributed around the country, with the majority of clubs being in the eastern region, fewer in the western region, and the fewest in the central region. They are mostly found along the eastern coast, which makes up around 57.0% of the entire region. According to the examination of the nearest neighbor index, Chinese Go League clubs are distributed spatially in a typically discrete way, with clustering and random distribution in individual years. It implies that little intercity influence among Chinese Go League clubs is insignificant.
- Chinese Go League clubs are geographically dispersed and grouped together, which benefits the development of the urban and regional Go League cultural environment, the flow of Chinese Go talent, the development of reserve talent, and the growth of the related sports and cultural industries. The relocation and spatial distribution of the Chinese Go League clubs’ host cities is discontinuous, with both centripetal agglomeration in the direction of significant cities and hierarchical diffusion outward from key cities. In contrast to the centripetal clustering of clubs in key cities, which will have a siphoning effect on talent and the economy, the spatial spread of clubs from key cities will undoubtedly result in talent migration, an increase in talent, and the development of Chinese Go culture and the related industries in the new cities, further strengthening the professionalization of the Chinese Go tournament in key cities like Beijing.
- Due to the frequent changes of sponsors and investors, the interaction between Chinese Go League clubs and host cities is varied and may be divided into three types: urban stability, urban transition, and urban fluctuation.

## Data Availability

The original contributions presented in the study are included in the article/Supplementary Material, further inquiries can be directed to the corresponding author.
